# Octaethyl vs Tetrabenzo Functionalized Ru Porphyrins on Ag(111): Molecular Conformation, Self-Assembly and Electronic Structure

**DOI:** 10.1021/acs.jpcc.4c06978

**Published:** 2024-12-16

**Authors:** Dennis Meier, Peter Knecht, Pablo Vezzoni Vicente, Fulden Eratam, Hongxiang Xu, Tien-Lin Lee, Alexander Generalov, Alexander Riss, Biao Yang, Francesco Allegretti, Peter Feulner, Joachim Reichert, Johannes V. Barth, Ari Paavo Seitsonen, David A. Duncan, Anthoula C. Papageorgiou

**Affiliations:** † Technical University of Munich, TUM School of Natural Sciences, Physics Department E20, Garching 85748, Germany; ‡ Diamond Light Source, Didcot OX11 0QX, U.K.; § MAX IV Laboratory, Lund University, Lund 22 484, Sweden; ∥ Département de Chimie, École Normale Supérieure (ENS), Paris 75005, France; ⊥ Centre National de la Recherche Scientifique, Université de Recherche Paris-Sciences-et-Lettres, Sorbonne Université, Paris 75005, France; # Laboratory of Physical Chemistry, Department of Chemistry, National and Kapodistrian University of Athens, Panepistimiopolis, Athens 157 71, Greece

## Abstract

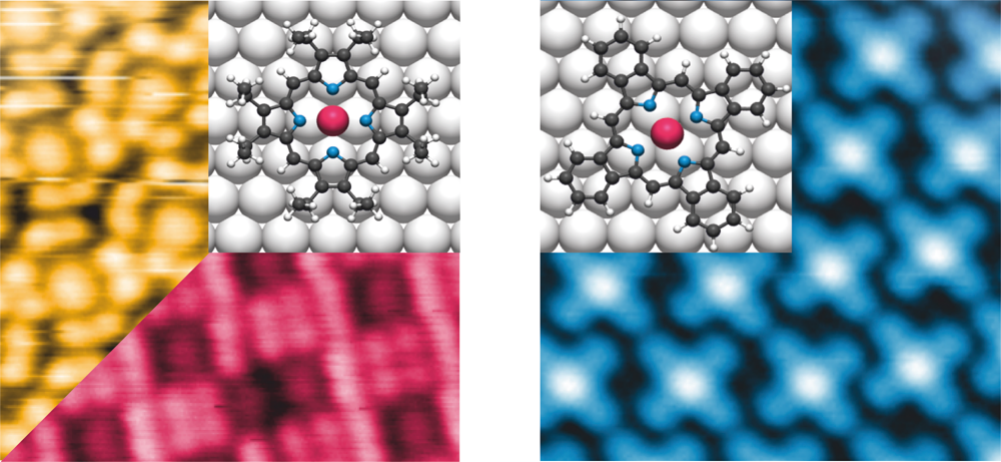

Metalloporphyrins on interfaces offer a rich playground for functional materials and hence have been subjected to intense scrutiny over the past decades. As the same porphyrin macrocycle on the same surface may exhibit vastly different physicochemical properties depending on the metal center and its substituents, it is vital to have a thorough structural and chemical characterization of such systems. Here, we explore the distinctions arising from coverage and macrocycle substituents on the closely related ruthenium octaethyl porphyrin and ruthenium tetrabenzo porphyrin on Ag(111). Our investigation employs a multitechnique approach in ultrahigh vacuum, combining scanning tunneling microscopy, low-energy electron diffraction, photoelectron spectroscopy, normal incidence X-ray standing wave, and near-edge X-ray absorption fine structure, supported by density functional theory. This methodology allows for a thorough examination of the nuanced differences in the self-assembly, substrate modification, molecular conformation and adsorption height.

## Introduction

Metalloporphyrins and their partially hydrogenated equivalents play a pivotal role in nature due to their high occurrence, the stabilization of various metal centers, and showcasing of diverse material properties. Advances in surface science techniques have spurred numerous research efforts to functionalize solid surfaces with porphyrins and investigate the properties of these interfaces.^1,2^ The expectations to explore novel materials have been stimulated by the ability of porphyrins to effectively stabilize a wide variety of elements including s-block,^3^ p-block,^4^ d-block^5−7^ and f-block^8−10^ elements in their cavities, which impart different functionalities. The capacity to tune the magnetic behavior^11−16^ or adsorption properties of small ligand molecules to the metal centers^17−23^ makes well-ordered interfaces of metalloporphyrins promising for potential applications in spintronics, gas sensing, and heterogeneous catalysis.

Changing the substituents of the macrocycle can influence the porphyrins’ functionalities.^24−31^ For example, iron tetrabenzo porphyrin (TBP) shows an increase in effective spin moment compared to iron octaethyl porphyrin (OEP) on Au(111),^27^ whereas Co-OEP has a spin magnetic moment on Cu(100), which is totally quenched for Co-TBP on the same substrate.^28^ Another effect of substituents on the functionality of porphyrins is seen in the adsorption of small molecules. While CO adsorbs on the metal center of ruthenium tetraphenyl porphyrin (TPP) on Ag(111), on the planarized Ru-TPP counterpart it does not do so under similar conditions.^31^ Hence, understanding the modifications introduced by different substituents on the porphin macrocycle is essential for tailoring the functionalities of metal porphyrin interfaces. On-surface ring-closure reactions are a viable way to modify the substituents of the porphyrins and phthalocyanines,^32−35^ e.g., by electrocyclic ring closure reactions of OEP and phthalocyanine precursors forming TBP or phthalocyanines, respectively.^32,34^

Here, we present a comprehensive study of the self-assembly and adsorption of Ru-OEP on Ag(111) as a function of molecular coverage under ultrahigh vacuum (UHV) conditions. We further show that on Ag(111), selective, intramolecular ring-closure reactions can modify the substituents of the Ru-OEP yielding exclusively Ru-TBP. This study aims to explore how these changes in substituents influence both the porphyrin macrocycle and the ruthenium metal center, as well as how molecular density can affect the porphyrin macrocycle. Ru-OEP and Ru-TBP exhibit variations in the chemical composition of their substituents, with Ru-OEP featuring ethyl side chains and Ru-TBP incorporating phenyl rings attached to the pyrroles of the porphyrin macrocycle. Scanning tunneling microscopy (STM), and low energy electron diffraction (LEED) were used to conduct real-space imaging of the self-assembly and analyze its periodicity in reciprocal space, respectively. X-ray photoelectron spectroscopy (XPS), ultraviolet photoelectron spectroscopy (UPS), and near-edge X-ray absorption fine structure (NEXAFS) provided insights into the chemical and electronic states of the different molecules on Ag(111). Furthermore, angle-dependent NEXAFS, combined with normal incidence X-ray standing waves (NIXSW), offered information on the out-of-plane positions of the molecules and their atoms. These experimental results are compared with relaxed structures of single molecules on Ag(111) derived from density functional theory (DFT) calculations. This comprehensive approach allows for a detailed characterization of three distinct ordered wetting layers of Ru porphyrins on Ag(111), which have not been reported previously.

## Methods

### Sample Preparation

The presented results were obtained using five different UHV systems (base pressure < 4 × 10^–10^ mbar). The single crystal Ag(111) surface was prepared in situ by Ar^+^/Ne^+^ sputtering followed by annealing to 725 K. After outgassing in UHV, Ru(CO)-OEP (Sigma-Aldrich) was deposited via organic molecular beam epitaxy (OMBE) by heating the crucible to 490–540 K with the Ag(111) surface at room temperature (rt). No CO molecules bonded to the metal center were detected on the surface by XPS. The high coverage preparations of Ru-OEP were achieved either by controlling the deposition time or by preparing a multilayer followed by annealing to 500 K. Ru-TBP was prepared by depositing Ru(CO)-OEP on Ag(111) kept at 700 K promoting ring closure of the ethyl side chains.^34^ Heating the sample with deposited Ru-OEP molecules resulted in multiple reaction products due to the loss of selectivity between intramolecular and intermolecular reactions of the ethyl side chains (Figure S1). The verification of the sample structure was performed by either STM or LEED prior to further analysis.

### STM

A variable temperature Aarhus STM (SPECS GmbH) in a custom-built UHV system was used to study the Ru-OEP interfaces at rt or at approximately 150 K. The STM images of Ru-TBP were taken at a commercial noncontact atomic force microscopy (nc-AFM)/STM system (CreaTec) operated at 6 K in a custom-built UHV system. For the data evaluation, SpmImageTycoon was used.^36^ Both instruments consist of a preparation chamber and an analysis chamber, with the STM housed in the latter, separated by a gate valve. Both STM systems applied the tunneling bias to the sample, used tungsten tips, and were located at Technical University of Munich (TUM, Germany). The tunneling parameters are given in the corresponding Figure caption.

### LEED

A commercial multichannel plate (MCP) LEED apparatus at the I09 beamline at the Diamond Light Source (DLS, U.K.) or a commercial LEED in custom-built UHV system at TUM both from OCI Vacuum Microengineering Inc. were used. The samples were at rt (DLS) or 90 K (TUM) during measurements. No temperature-dependent differences in the LEED pattern were observed. The LEED patterns were simulated using the LEEDpat software (https://www.fhi.mpg.de/958975/LEEDpat4) by K. Hermann and M. A. Van Hove.

### NEXAFS

The NEXAFS measurements were taken at the FlexPES end station in MAX IV Laboratory in Lund (Sweden).^37^ The C and N K-edges were measured at 200 K with partial electron yield (PEY) detection by an in-house-built MCP detector with a retardation grid voltage of 250 and 350 V, respectively. For all systems, five different incidence angles (θ = 30, 45, 60, 75 and 90°) between the surface normal and the **E** vector of the linearly polarized light (polarization, P, of 90%) were measured. At least three spectra for each angle were taken. Evaluation of the NEXAFS data sets was performed by well-established standard procedures.^38,39^ The process involved subtracting the bare crystal signal from the sample spectrum, subsequently correcting for photon flux, and normalizing the edge jump to a value of one. Symmetrical and asymmetrical gaussian lineshapes were used to fit the spectra. The formula for a 3-fold symmetry or higher was used to determine the tilt angle of the adsorbed molecules.^38^

### NIXSW

The NIXSW measurements were performed in the permanently mounted endstation in EH2 of beamline I09 in DLS.^40^ All samples were measured at 200 K, using a Scienta EW4000 HAXPES analyzer, oriented perpendicularly to the incident X-rays in the horizontal plane of the photon linear polarization. The measurements for the (111) planes, parallel to the surface, were performed at a normal-incidence Bragg energy of *h*ν = 2.63 keV. Multiple repetitions of measurements were conducted at different spots on the sample. At each spot, the reflectivity curve was measured to precisely align the energy for individual NIXSW measurements and ensure the crystalline quality of Ag(111). To monitor potential beam damage, XP spectra of the C 1s and Ru 3d regions were recorded before and after each NIXSW measurement.

### XPS/UPS

The XP and UP spectra were recorded at the end station I09 in DLS, using photon energies of 550 eV (N 1s core level), 450 eV (Ru 3d and C 1s core levels) and 135 eV (valence band). The binding energy was calibrated with the corresponding Fermi edge measured at the same photon energy. For the fits of the C 1s core level, a Shirley background was subtracted, and Voigt functions were employed to fit the individual peaks. The here presented spectra of the N 1s core level and the valence band are not processed.

### Work Function

The work function was determined for a Ru-OEP or Ru-TBP covered crystal by secondary electron cutoff measurements^41^ by a standard Al Kα source and a SPECS Phoibos 100 CCD hemispherical analyzer in normal emission geometry in a custom-built UHV system at TUM.

### DFT

DFT geometry optimization were performed with the Quantum ESPRESSO package.^42^ The rB86-vdW-DF2 approximation was utilized for the exchange–correlation term,^43,44^ taking into account five layers of the silver substrate, with the two lowest layers held in their bulk-terminated positions. The optimization parameters included an optimized lattice constant of 4.1075 Å, a 2 × 2 *k*-point mesh, Fermi–Dirac smearing of occupation numbers with a 50 meV broadening, and projector augmented wave (PAW)^45^ data sets for the pseudization of the core electrons. Surface-dipole corrections were applied, and the cutoff energy was set to 60 Ry for the wave functions and 600 Ry for the electron density. For both porphyrins, a single molecule was optimized in a rectangular unit cell with dimensions of 7 × 4√3 (comprising seven unit cells along the high-symmetry direction and four double-rows perpendicular to it), resulting in lattice vector lengths of 20.331 and 20.123 Å, respectively.

## Results and Discussion

We examined three systems on Ag(111) to address the questions posed. Two distinct self-assemblies of Ru-OEP were identified: a lower coverage relaxed phase and a higher coverage compressed phase. For Ru-TBP, only a single phase was observed and investigated. Throughout this paper, these systems are distinguished using the following color code: yellow for the relaxed phase of Ru-OEP, pink for the compressed phase of Ru-OEP, and blue for Ru-TBP.

### Scanning Tunneling Microscopy

**Figure 1:**
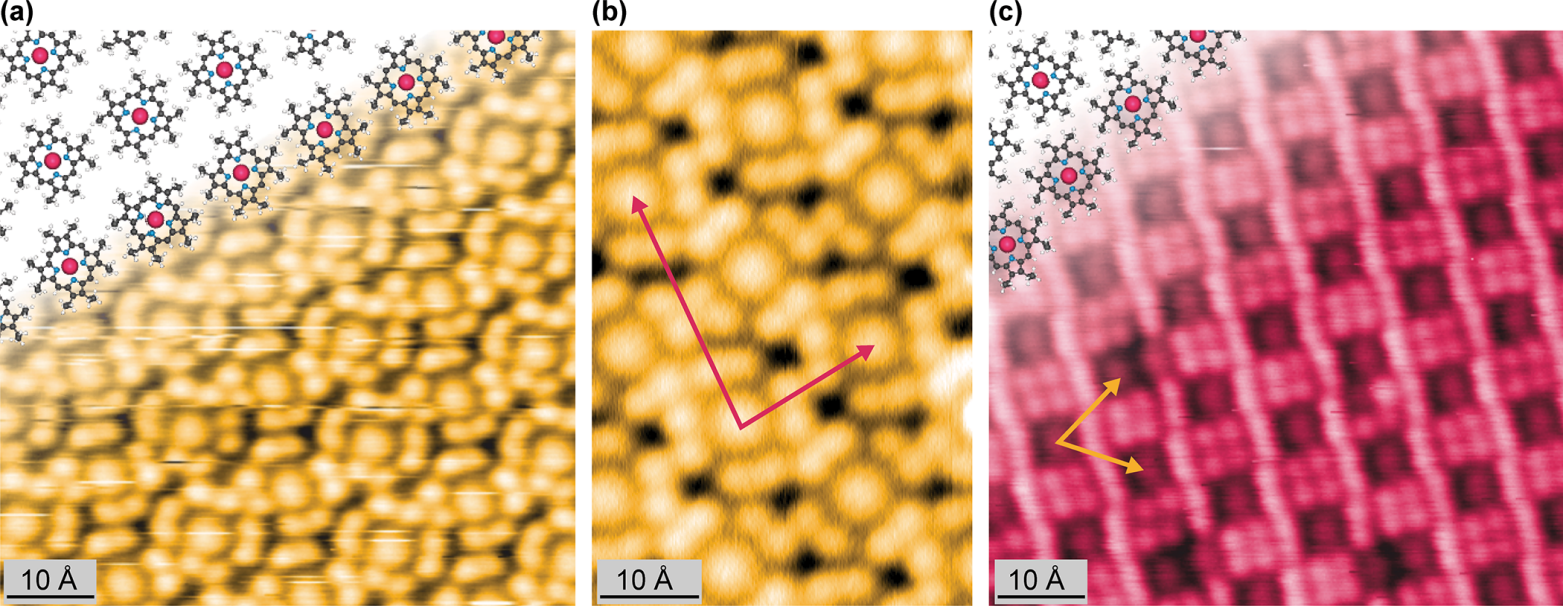
STM images of Ru-OEP on Ag(111). (a,b) Relaxed phase (a: 40 pA, 525 mV, 170 K; b: 40 pA, 300 mV, 5 K). (c) Compressed phase (20 pA, 1250 mV, rt). To visualize the self-assembly, molecular models are overlaid with C, N, H, and Ru atoms depicted in gray, blue, white, and pink, respectively. The LEED derived unit cell vectors are marked in pink (b) and yellow (c) (see also Figures S2 and S4, respectively).

First, we will discuss the deposition of Ru-OEP on Ag(111). A distinct molecular packing is observed for surfaces with bare Ag(111) patches. This phase is referred to as the relaxed phase in this paper and can completely cover the Ag(111) surface (as verified with STM). In these self-assembled islands, individual molecules can be recognized (Figure 1a). They exhibit a central protrusion encircled by four pairs of smaller protrusions appearing with similar apparent height at a bias of 525 mV. The central feature is assigned to the porphyrin macrocycle, while the eight small protrusions are ascribed to the ethyl side chains.^5,46,47^ Two distinct molecular orientations can be discerned, differentiated by a rotation of 31 ± 2° within the surface plane (Figure 1a). This variation is significantly larger than the reports for other OEPs on single-crystal low-index metal surfaces (0 to 15°).^5,47−49^ The axis through two opposite *meso* carbons in both orientations of the Ru-OEP aligns with a high symmetry axis of the Ag(111) substrate. Consequently, we attribute the observed higher difference in rotation within the same domain to interactions with the substrate. A well-ordered self-assembly is formed by rows of alternating molecules (Figure 1a). Consequently, the surface unit cell comprises two molecules. Based on LEED, the unit cell is described by the commensurate epitaxy matrix of  (24.7 Å × 15.3 Å, Φ = 84.9°, Figure S2) marked in Figure 1b. Hence, the islands of the relaxed phase have a molecular density of 5.3 × 10^–3^ molecules · Å^–2^.

By depositing more Ru-OEP onto a surface fully covered with the relaxed phase, a more densely packed phase was observed (Figure 1c), hereinafter referred to as compressed phase. The occurrence of a separate, high coverage phase was also reported for Ru-TPP on Ag(111), where it was ascribed to the high affinity of Ru to the Ag(111) surface.^50^ It was rationalized that the energy gain from the adsorption of more Ru-porphyrins must be greater than the energetic penalty of distorting the adsorption geometry away from the relaxed geometry. The difference of the Ru-OEP adsorption geometry from the relaxed phase to the compressed phase is addressed by the NIXSW investigation (vide infra).

The intermolecular contrast of Ru-OEP is strongly bias dependent. At a bias of 1250 mV we see the ethyl chains brighter compared to the central porphyrin ring (Figure 1c). A similar contrast change is noticeable for negative biases (Figure S3). The individual molecules show the same orientation with respect to the high symmetry axis as in the relaxed phase. However, no rotation of the adjacent porphyrins in the compressed phase could be observed and the unit cell comprises one molecule with an overlayer matrix of  (13.7 Å × 13.8 Å, Φ = 65.2°, Figure S4) derived by LEED. The unit cell excludes a single adsorption site of the molecules within the layer. The molecular density is 5.8 × 10^–3^ molecules · Å^–2^, higher than for the relaxed phase.

**Figure 2:**
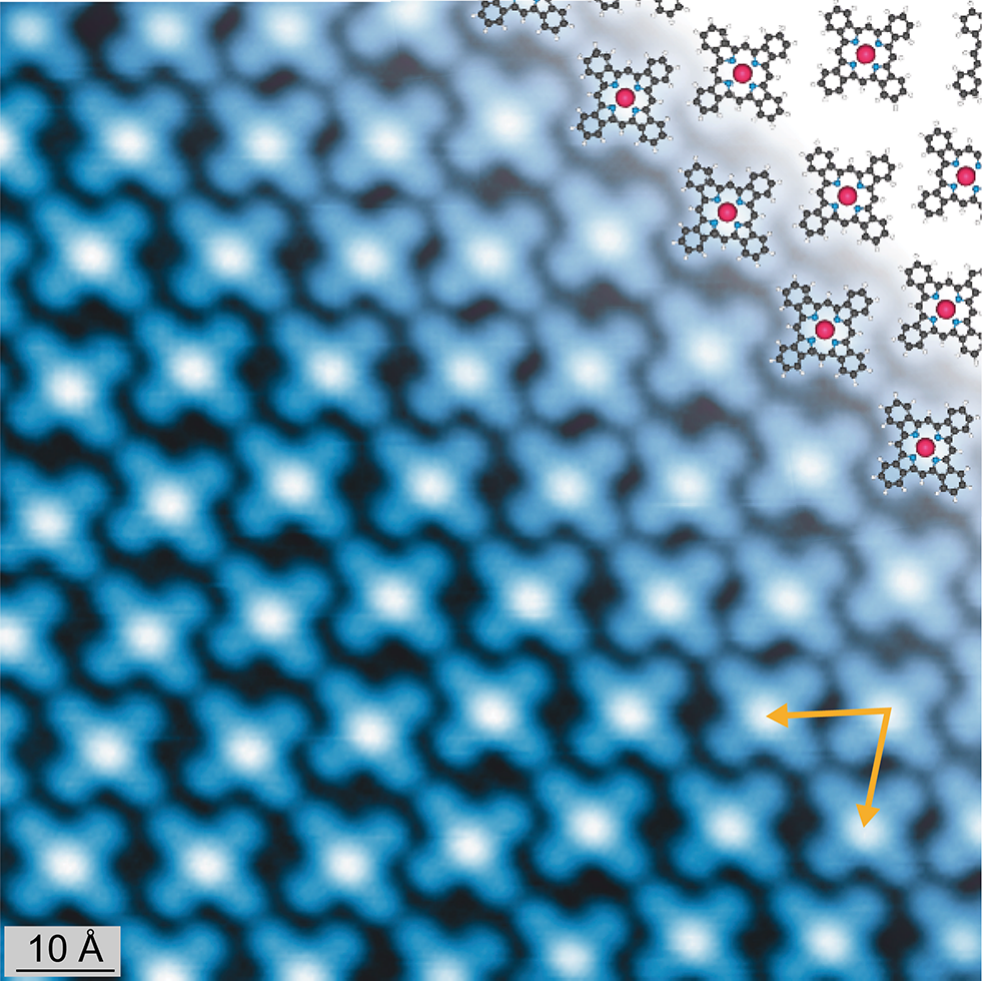
STM image of a monolayer of Ru-TBP (50 pA, 200 mV, 6 K) on Ag(111). The derived LEED unit cell vectors are marked in yellow (Figure S7). To visualize the self-assembly, molecular models are overlaid with C, N, H, and Ru atoms depicted in gray, blue, white, and pink, respectively.

As another presumably flat ruthenium porphyrin, Ru-TBP was prepared by means of on-surface synthesis (vide supra) to investigate the impact of the substituents on organization and the Ru center within the macrocycle. At a low coverage, individual molecules were discerned in STM (Figure S5). Upon increasing the coverage of Ru-TBP, self-assembled islands formed (Figure 2). The contrast of a single molecule is characterized by a central protrusion surrounded by four smaller protrusions on each side. The latter is assigned to the newly formed phenyl rings, comparable to other metallo TBPs and phthalocyanines.^32,34^ The contrast of Ru-TBP exhibits a bias dependency, with the center more pronounced at lower biases and the phenyl rings dominating at higher biases (Figure S6). The overlayer matrix of  (14.1 Å × 13.7 Å, Φ = 73.4°) describes the unit cell detected by LEED (Figure S7). This yields a molecular density of 5.2 × 10^–3^ molecules · Å^–2^. Discrepancies between the LEED derived unit cell and the STM images can be observed (Figure S8) and may be tentatively attributed to the different acquisition temperatures and, possibly, to different layer strain. The LEED data was taken at 200 K, whereas the STM images were recorded at 5 K. As LEED is a space-averaging technique, mobility of the Ru-TBP molecules can lead to different results, whereas in the STM images all mobility is frozen.

### X-ray and Ultraviolet Photoelectron Spectroscopy

**Figure 3:**
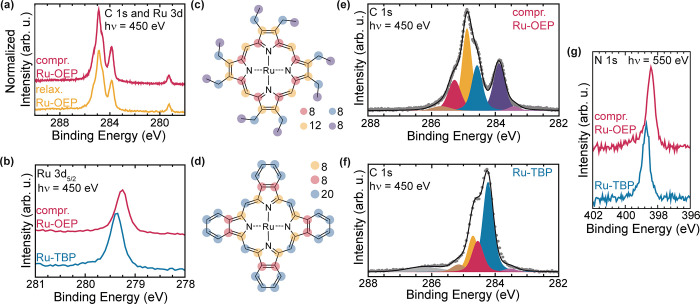
(a) Normalized XP spectra of the C 1s and Ru 3d region of the relaxed phase (yellow) and the compressed phase (pink) of Ru-OEP (Fewer scans were taken for the compressed phase spectrum, and it was offset for better comparability.). (b) Ru 3d_5/2_ core level of compressed phase Ru-OEP (pink) and Ru-TBP (blue) on Ag(111). Molecular model of (c) Ru-OEP and (d) Ru-TBP. Fitted C 1s XP spectra of (e) compressed phase of Ru-OEP and (f) Ru-TBP on Ag(111). The fitted peaks are colored the same as the corresponding carbons in the respective molecular model; the overlapping Ru 3d_3/2_ component is highlighted in bright purple. (g) XP spectra of the N 1s region of the compressed phase of Ru-OEP (pink) and Ru-TBP (blue) on Ag(111).

The chemical state of the ruthenium porphyrins was analyzed via XPS. Analysis of the XP spectra for both the relaxed and compressed phases of Ru-OEP revealed no apparent differences (Figure 3a), except in intensity. Consequently, we focus here on the description of the compressed phase, which gives a stronger signal. The Ru 3d_5/2_ core level was chosen to assess the chemical state of the Ru metal center (Figure 3b), since the Ru 3d_3/2_ peak overlaps with the C 1s peak envelope (Ru 3d has a spin orbit splitting of 4.2 eV).^51^ The binding energy of the Ru 3d_5/2_ peak is 279.2 eV. This value corresponds to metallic ruthenium^52^ rather than the expected binding energy for the oxidation state of ruthenium +2 shown in, e.g., a multilayer of Ru porphyrins.^50,53^ This behavior is comparable to other Ru porphyrin species on Ag(111).^50^ The shift can be partially ascribed to final state screening effects of the metal substrate.^54−56^ Nonetheless, the binding energy indicates a substantial charge transfer from the substrate to the Ru and, thus, a chemisorption of the ruthenium porphyrins. Further discussion of this phenomenon will follow in subsequent sections.

The XPS of the C 1s core level of Ru-OEP shows a distinct peak at 283.9 eV (purple, Figure 3e). This peak is assigned to the terminal –CH_3_ carbons of the side chains (purple, Figure 3c). It amounts to 23.7% of the total peak intensity, which fits well with the expected proportion of 22.2% (8 out of 36 atoms). The three other major peaks in at higher binding energy Figure 3e are assigned as follows: aliphatic carbons (—CH_2_—) of the ethyl side chains (blue, 284.5 eV), sp^2^-hybridized carbons (yellow, 284.9 eV), and α-pyrrole carbons with a bond to a nitrogen atom (pink, 285.3 eV). In addition, a weak component has to be attributed to the Ru 3d_3/2_ core level (light purple, 283.4 eV) and the higher binding energy peak is assigned to a C 1s shakeup satellite (brown, 285.7 eV).^56^Table 1 summarizes the fitted components and their intensities. The small deviations of their relative intensities in comparison to the stoichiometric ratio presumably reflect varying attenuations and possible effects of photoelectron diffraction by the metal substrate.

**Table 1:** Assignment of the Fitted Peaks of the C 1s XP Spectra of Ru-OEP and Ru-TBP from Figure 3, Along with Their Respective Binding Energies^a^

component	binding energy (eV)	#C by fit	#C in molecule
compressed phase Ru-OEP on Ag(111)			
—C—N	285.3	6.3	8
sp^2^-hybridized C	284.9	13.0	12
—CH_2_—	284.6	8.6	8
—CH_3_	283.9	8.1	8
Ru-TBP on Ag(111)			
—C—N	284.7	19.6	20
sp^2^ C with 3 C—C bonds	284.5	7.9	8
sp^2^ C with 2 C—C bonds	284.2	8.5	8

a The number of carbon atoms in the examined molecule derived from the percentage of the fitted peak area and the corresponding actual number are listed in columns 3 and 4, respectively.

**Table 2:** Primary Peak Assignments for the N 1s Peaks in the Compressed Phase Ru-OEP on Ag(111) and Ru-TBP on Ag(111) NEXAFS Spectrum Below Ionization Energy

experimental peak positions (eV)	transition
compressed phase Ru-OEP on Ag(111)	
399.0	(1s) → π*
401.4	(1s) → π*
401.9	(1s) → σ*
403.0	(1s) → π*
Ru-TBP on Ag(111)	
399.2	(1s) → π*
400.4	(1s) → π*
402.0	(1s) → σ*
402.3	(1s) → π*
403.9	(1s) → π*

The XPS of Ru-TBP reveals a similar shift to lower binding energies (compared to spectra from multilayer films) of the Ru 3d_5/2_ peak as observed in Ru-OEP (Figure 3b), suggesting a similarly strong interaction with the substrate. The peak in Ru-TBP is shifted by only 0.1 eV to higher binding energies, with respect to Ru-OEP. However, a strong change in the C 1s core level shape can be observed. Ru-TBP has a narrower C 1s signal due to the increase in the proportion of carbon atoms that are sp^2^ hybridized, making the chemical state of all carbon atoms more similar than in Ru-OEP (Figure 3d,f). Furthermore, the distinct peak of the ethyl side chains is no longer observable. This indicates the completion of the intramolecular ring closure reactions. Accordingly, the carbon signal was fitted by three distinct peaks (Figure 3f, Table 1), which are attributed to α-pyrrole carbons with a bond to nitrogen (yellow, 284.7 eV), sp^2^ hybridized carbons with three C–C bonds (pink, 284.5 eV) and carbons with two C–C bonds (blue, 284.2 eV) (Figure 3d,f). A broad peak at a binding energy of 286.2 eV is assigned to a low amount of highly oxidized carbon species, which presumably formed during the deposition of Ru-OEP on Ag(111) at 700 K. Only one peak is observable in the N 1s core level region for both porphyrins due to the chemical equivalence of all four nitrogen atoms in the molecules (Figure 3g). However, the peak in Ru-TBP is shifted by 0.3 eV to higher binding energies compared to the Ru-OEP peak.

**Figure 4:**
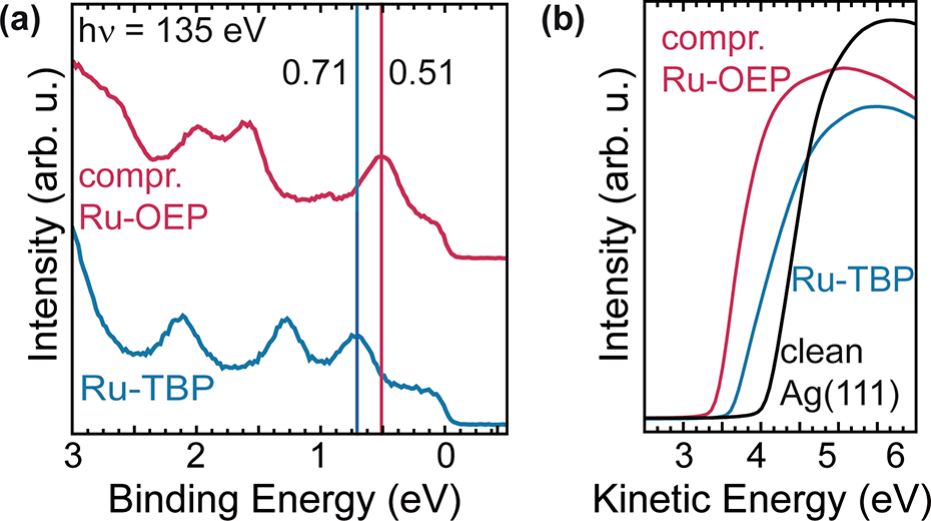
(a) Valence band of compressed phase Ru-OEP (pink) and Ru-TBP (blue) on Ag(111). The spectra are offset vertically for clarity. (b) XPS secondary electron cutoff measurements of the compressed phase Ru-OEP (pink) and Ru-TBP (blue) functionalized Ag(111) surface and a clean Ag(111) surface (black) used to determine the respective work function values.

The valence band spectra of Ru-OEP and Ru-TBP reveal significant differences between the two Ru porphyrins (Figure 4a). In both molecules, a state below 1 eV with respect to the Fermi edge is observed, indicative of charge transfer from the substrate to the Ru 4d orbitals, as found before for other metalloporphyrins.^54,57^ This state agrees well with the bright contrast of the porphyrin macrocycle at low negative biases, but differs by 0.2 eV for the two different porphyrins. Ru-TPP on Ag(111) shows a similar state at an even lower binding energy of 0.4 eV.^31^ Ru-TBP as well as Ru-OEP exhibit two further states, which differ in their positions relative to each other at 1.3 eV (1.6 eV) and 2.1 eV (2.0 eV) for Ru-TBP (Ru-OEP). The significant differences in the valence band spectra between Ru-OEP and Ru-TBP on Ag(111) are in stark contrast to Co-OEP and Co-TBP on Ag(100), which show only minor differences.^30^

We further used photoelectron spectroscopy to determine the work function of these interfaces, a property which, e.g., significantly influences the catalytic activity of surfaces.^58,59^ The work functions of the compressed phase Ru-OEP/Ag(111), Ru-TBP/Ag(111) and pristine Ag(111) were determined by recording the photoemission secondary electron cutoff (Figure 4b). A change of −0.63 eV (absolute: 3.89 eV) for Ru-OEP and of −0.42 eV (absolute: 4.11 eV) for Ru-TBP with respect to the clean Ag(111) was observed.

### Near Edge X-ray Absorption Fine Structure

**Figure 5:**
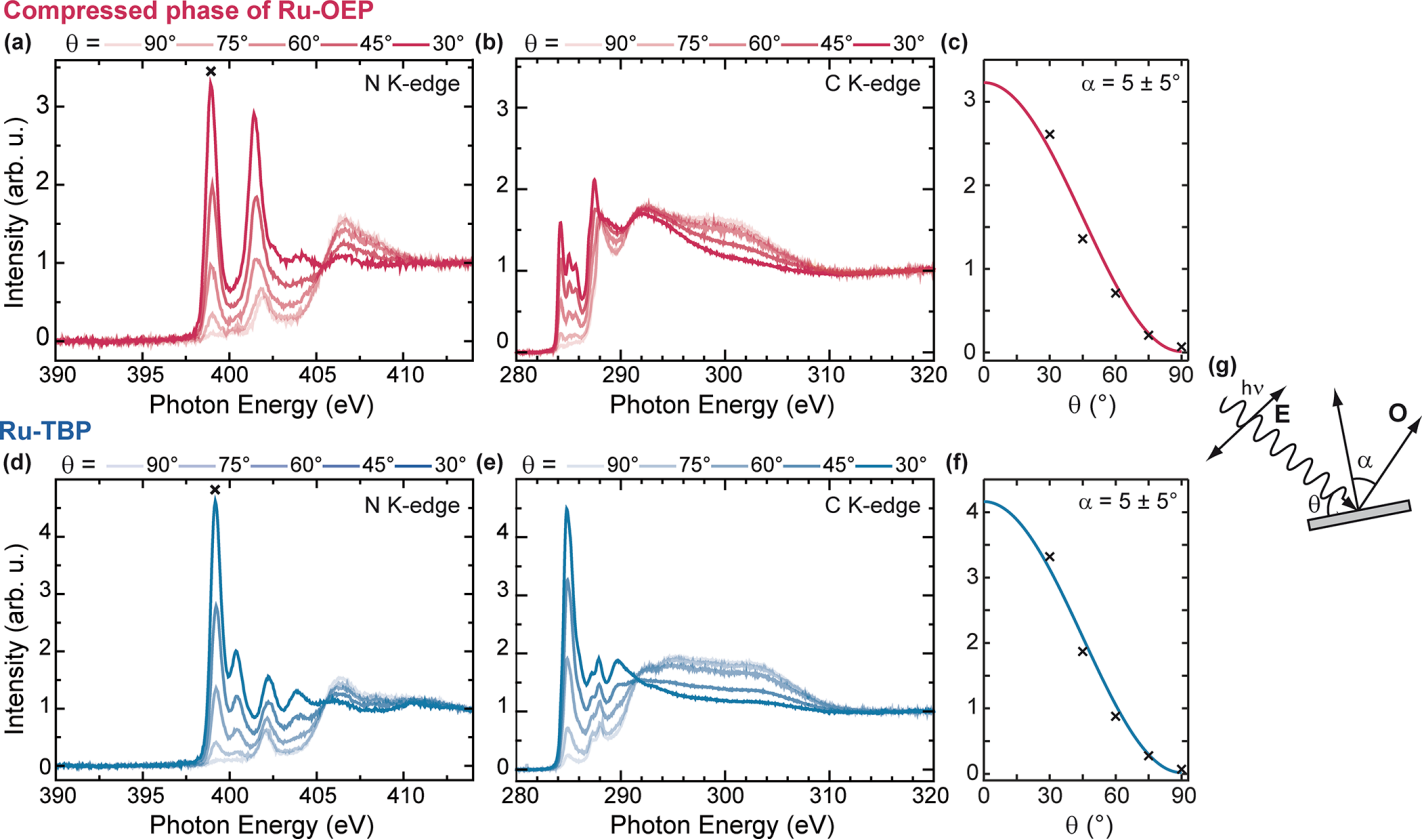
(a) N and (b) C K edge NEXAFS measurements of the compressed phase of Ru-OEP on Ag(111) acquired at five different angles of photon incidence. (c) Curve fitting analysis of the π* resonance indicated by a cross mark in (a). (d) N and (e) C K edge NEXAFS measurements of Ru-TBP on Ag(111) acquired at five different angles of photon incidence. (f) Curve fitting analysis of the π* resonance denoted, by a cross mark in (d). (g) Schematics of the beam-sample-geometry with the electric field vector **E** of the incoming X-ray radiation and the final state orbital direction of maximal amplitude **O** and their angles θ and α to the surface or surface normal, respectively.

Angular dependence N K-edge and C K-edge NEXAFS measurements were conducted to gain insights into the unoccupied states of the porphyrins and the orientation of its pyrrole rings relative to the substrate (Figure 5a,b,d, and e). N K-edge NEXAFS measurements of Ru-OEP/Ag(111) show four distinct resonances below the adsorption edge: two major resonances at photon energies of 399.0 and 401.4 eV and two minor resonances at 401.9 and 403.0 eV (Figure 5a). All resonances show a strong dichroism. However, the resonance at 401.9 eV has the opposing dichroism with respect to the other transitions. We assign it to a σ* resonance attributed to a mixed ligand Ru antibonding orbital (Table 2).^30,60^ The other resonances are assigned to π* transitions (Table 2). The N K-edge of Ru-TBP shows five resonances below the ionization energy: a dominant resonance at 399.2 eV and four minor resonances at 400.4, 402.0, 402.3, and 403.9 eV (Figure 5d). Here again, one resonance (402.0 eV) shows an opposite dichroism than the other and is therefore attributed to σ* resonance to a mixed ligand Ru antibonding orbital (Table 2).^30,60^ The other four peaks are attributed to π* transitions (Table 2).

The pronounced dichroism in the π* region of the N K-edge of the compressed phase of Ru-OEP (Figure 5a) shows its maximal intensity when the electric field of the linear polarized X-rays is almost perpendicular to the surface (θ = 30°, Figure 5g). Conversely, when the electric field of the linear polarized X-rays is aligned parallel to the surface, the π* region is suppressed. The C K-edge shows a similar dichroism (Figure 5b). Similar observations were made for the relaxed phase of Ru-OEP (Figure S9). For a quantitative estimation of the orientation of the porphyrin macrocycle, the angular dependence NEXAFS intensities were analyzed. The best fit of the π* resonance peak at 399.0 eV is shown in Figure 5c and corresponds to a tilt angle of 5 ± 5° (Figure 5g). The C and N K-edge NEXAFS spectra of Ru-TBP exhibit a similar strong dichroism in the π* region as seen for the Ru-OEP (Figure 5d,e) and are also reported for other tetrabenzo porphyrin and phthalocyanine molecules.^30,61^ An analogous evaluation of the angular dependence π* resonance intensities of the peak at 399.2 eV was conducted (Figure 5f) and yielded a similar macrocycle tilt angle of 5 ± 5° (Figure 5g). Therefore, we can infer that both porphyrins are adsorbed in a predominantly parallel orientation to the surface, similar to other OEP and TBP species,^22,30,62^ but unlike Ru-TPP on Ag(111) which exhibits a strong saddle shape conformation of its macrocycle with α ∼ 30°.^63^

### Normal Incidence X-ray Standing Waves

**Figure 6:**
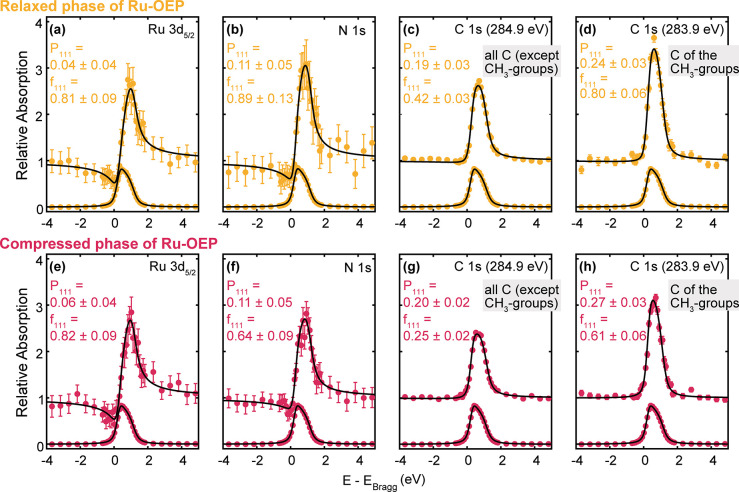
(111) NIXSW data of (a–d) the relaxed phase and (e–h) the compressed phase of Ru-OEP on Ag(111), respectively. The derived coherent position and coherent fraction are shown in the corresponding spectrum. The assignments of the binding energy of the two C 1s core level peaks are derived from XP spectra labeled in the respective fit.

**Figure 7:**
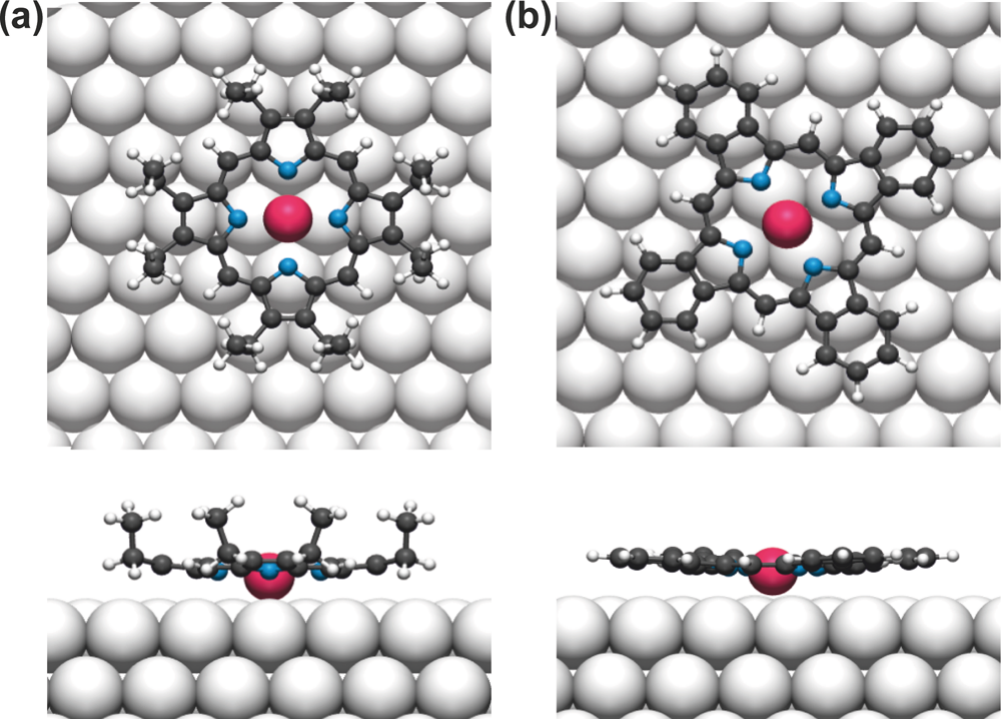
Top and side views of DFT optimized structure of (a) isolated Ru-OEP and (b) isolated Ru-TBP on Ag(111). C, N, H, Ru, and Ag atoms are represented by gray, blue, small white, pink, and large white spheres, respectively.

To gain deeper insights into the out-of-plane positions of the atoms, we recorded the NIXSW absorption profiles from the N 1s, C 1s, and Ru 3d core levels at the (111) reflection of the Ag substrate (Table 3). The Ru 3d_5/2_ profile of the relaxed Ru-OEP phase measures an adsorption height of 2.45 ± 0.09 Å (P_111_ = 0.04 ± 0.04) and, with a coherent fraction of 0.81 ± 0.09, which is indicative of a single adsorption height (Figure 6a).^64^ Within the error, the experimental adsorption heights match with the DFT derived adsorption height of 2.44 Å of the Ru metal center of a single relaxed Ru-OEP molecule on Ag(111) (Figure 7a and Table 3) and with the reported adsorption height of planarized Ru-TPP on Ag(111) (2.45 ± 0.02 Å).^50^ The proximity of the metal center to the surface strongly suggests a robust chemisorption. The N 1s profile of the relaxed Ru-OEP phase indicates an adsorption height of 2.62 ± 0.12 Å (P_111_ = 0.11 ± 0.05) with a high coherent fraction of 0.89 ± 0.13 (Figure 6b). Thus, the Ru atom is placed between the Ag(111) plane and the N atom plane as reported for Ru-TPP and its planarized derivates.^50^

**Table 3:** Summary of the Coherent Fraction (f_111_) and Position (P_111_) Derived by NIXSW^a^

component	P_111_	f_111_	adsorption height (Å)	
			NIXSW	DFT
relaxed phase Ru-OEP				
Ru 3d_5/2_	0.04 ± 0.04	0.81 ± 0.09	2.45 ± 0.09	2.44
N 1s	0.11 ± 0.05	0.89 ± 0.13	2.62 ± 0.12	2.68
C 1s (284.9 eV)	0.19 ± 0.02	0.42 ± 0.03	2.81 ± 0.05	3.00
C 1s (283.9 eV)	0.24 ± 0.03	0.80 ± 0.06	5.26 ± 0.07	4.79

compressed phase Ru-OEP				
Ru 3d_5/2_	0.06 ± 0.04	0.82 ± 0.09	2.50 ± 0.09	2.44
N 1s	0.11 ± 0.05	0.64 ± 0.09	2.62 ± 0.12	2.68
C 1s (284.9 eV)	0.20 ± 0.02	0.25 ± 0.02	2.83 ± 0.05	3.00
C 1s (283.9 eV)	0.27 ± 0.03	0.61 ± 0.06	5.33 ± 0.07	4.79

Ru-TBP				
Ru 3d_5/2_	0.10 ± 0.05	0.94 ± 0.13	2.59 ± 0.12	2.56
N 1s	0.15 ± 0.06	0.80 ± 0.13	2.69 ± 0.12	2.75
C 1s (284.6 eV)	0.17 ± 0.07	0.41 ± 0.11	2.75 ± 0.14	2.90
C 1s (284.2 eV)	0.31 ± 0.04	0.66 ± 0.09	3.08 ± 0.09	3.11

a The experimental adsorption height was determined with an assumed (111) *d* spacing of 2.35 Å. Note that the given adsorption heights for species with a coherent fraction below 0.75 cannot be assumed to correspond to a uniform adsorption height.^64^

For the fit of the C 1s core level of the relaxed phase, two peaks were used–one at 283.9 eV for the methyl end groups and one peak at 284.9 eV corresponding to the other carbon species derived from XPS (Figure 6c,d). The coherent position of the peak at 283.9 eV in the relaxed phase of Ru-OEP, which corresponds to the CH_3_ species (Figure 3e), shows a high coherent fraction of 0.80 ± 0.06 (P_111_ = 0.24 ± 0.03), which hints to a uniform adsorption height of the side chains.^64^ The C 1s core level peak at 284.9 eV, which corresponds to all other C atoms in the Ru-OEP molecules (pink, yellow and blue components in Figure 3e), has a low coherent fraction of 0.42 ± 0.03 (P_111_ = 0.19 ± 0.02). Hence, no single adsorption height can be determined, which is expected as this peak relates to multiple different carbon species. An upward orientation of the ethyl side chains is reported for several OEPs on different substrates^5,24,30,47,65,66^ and aligns well with our DFT optimization of a single Ru-OEP molecule on Ag(111) (Figure 7a). This agrees well with the bias dependent contrast seen in STM, where, at high bias, the ethyl side chains are far more prominent than the center, supporting higher-lying ethyl side chains with respect to the porphyrin core. Therefore, we deduce an upstanding orientation and allocate an adsorption height for the CH_3_ species of 5.26 ± 0.07 Å derived by NIXSW.

For Ru-OEP in the relaxed phase, a perfectly planar conformation of the macrocycle can be excluded, due to the low coherent fraction (f_111_ = 0.42 ± 0.03) of the C 1s signal associated with the macrocycle and the 8 – CH_2_– carbons (284.9 eV). Different distortions of porphyrins’ or phthalocyanines’ macrocycles are reported as saddle-shape, bowl-shape, or a vertical offset model.^18,50,61,67^ The high coherent fraction of the nitrogen and the low coherent fraction of the macrocycles’ carbon atoms, along with the lower average adsorption height of the nitrogen compared to the carbon, can be rationalized by a bowl-like distortion in the porphyrin macrocycle. A saddle shape distortion would contradict the high coherent fraction of the methyl end groups and their higher adsorption height compared to the macrocycle. The relaxed DFT structure further supports the bowl-shape conformation (Figure 7a and Table 3).

The compressed phase shows a high coherent fraction of 0.82 ± 0.09 (P_111_ = 0.06 ± 0.04) in the NIXSW profile of the Ru 3d_5/2_ and a reduction of the coherent fractions of C 1s and N 1s core levels compared to the relaxed phase (Figure 6f–h). The terminal methyl groups’ peak has a coherent fraction of 0.61 ± 0.06, which excludes a single adsorption height. Distinct variations are also discernible within the structure in STM, as evident by the contrast difference of the ethyl group features with the same orientation toward the scanning direction (Figure 1c). This indicates subtle alterations in their respective heights. The reduction of the coherent fraction can also be observed on the other carbon peak and the nitrogen peak. However, the similarity in coherent positions to the relaxed phase of Ru-OEP indicates a resemblance in the average conformation of Ru-OEP in both phases. Therefore, a single adsorption height is deduced for the molecules in the compressed phase and the loss in coherent fraction is ascribed to a stronger distortion in the porphyrin macrocycle and substituents. On top, small deviations from the porphyrins’ optimal conformation might arise due to the reduction of the molecular footprint on the surface. In conclusion, a transition from the relaxed to the compressed phase of Ru-OEP has an impact on the porphyrin ring, but the metal center is not influenced.

**Figure 8:**
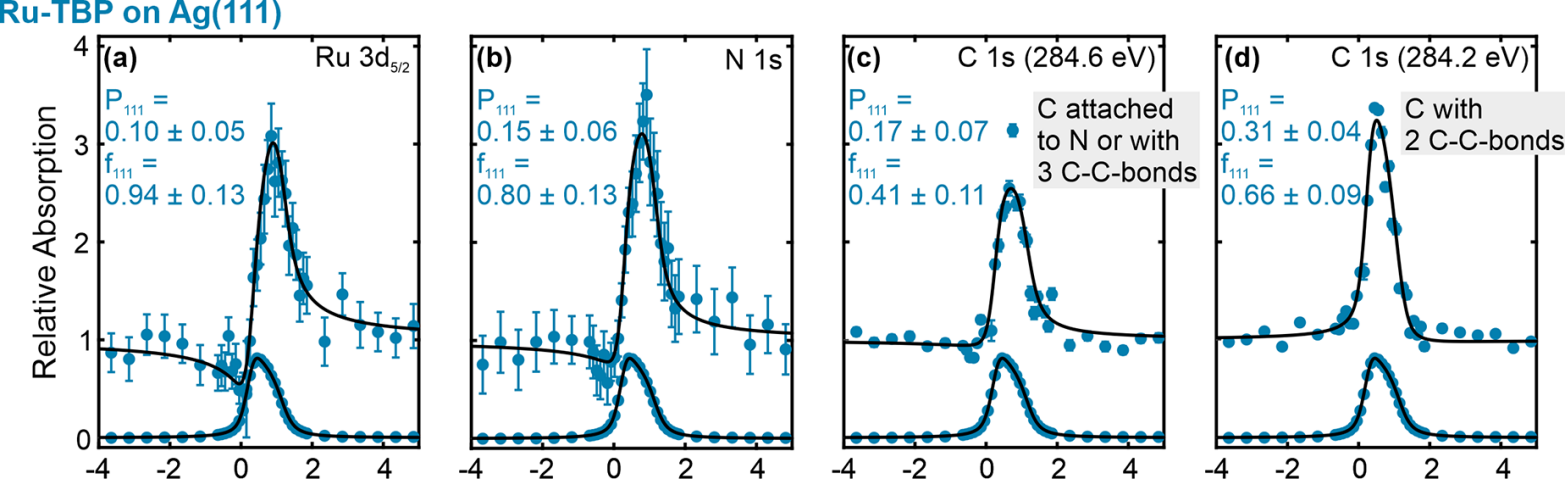
(a–d) (111) NIXSW data of Ru-TBP on Ag(111). The coherent position and coherent fraction extracted by the fits are given in the respective spectrum. The assignments of the binding energy of the two C 1s core level peaks are derived from XP spectra labeled in the respective fit.

The Ru 3d_5/2_ profile of Ru-TBP shows a slightly elevated adsorption height of 2.59 ± 0.12 Å (f_111_ = 0.94 ± 0.13) compared to Ru-OEP (Figure 8a). Considering the high coherent fraction, we assume a singular adsorption height for the Ru metal center. This slightly elevated adsorption height relative to Ru-OEP is also seen with DFT (Figure 7b and Table 3). The N 1s profile also exhibits a high coherent fraction (f_111_ = 0.80 ± 0.13) with a slightly higher coherent position (P_111_ = 0.15 ± 0.06) in comparison to the Ru 3d_5/2_ (Figure 8b and Table 3). We therefore infer a uniform adsorption of the Ru–N4 center parallel to the surface. The slightly higher adsorption height suggests a reduced core-hole screening of the Ru-TBP by the metal substrate compared to Ru-OEP. This difference can lead to the observed slightly higher binding energies of the N 1s and Ru 3d_5/2_ core levels in XPS (Figure 3b,g), as well as in the valence band in UPS (Figure 4a). A two-component fit was employed for the C 1s core level, with one component at 284.2 eV for the carbon with two C–C bonds and another at 284.6 eV for the carbons with bonded to nitrogen and the carbons with three C–C bonds (Figure 8c,d). The positions of the fitted NIXSW peaks were chosen according to the C 1s XP spectrum. The carbon atoms associated with two C–C bonds exhibit a coherent fraction of 0.66 ± 0.09, situated at an average height of 3.08 ± 0.09 Å (P_111_ = 0.31 ± 0.04). On the other hand, the remaining carbon species display a lower position (P_111_ = 0.17 ± 0.07) and a reduced coherent fraction (f_111_ = 0.41 ± 0.11). It is noteworthy that this peak comprises two distinct carbon species; thus, a lower coherent fraction is anticipated, if the porphyrin’s macrocycle has a nonflat conformation. These adsorption heights are well reproduced by the relaxed DFT structure (Table 3). In a saddle shape conformation, we would expect a lower coherent fraction of the carbons in the phenyl ring, since two of them bent upward and two downward, which is not observed. Thus, we assume a bowl shape conformation. This can explain the lower position of the porphyrin macrocycle compared to the phenyl rings and the high coherent fraction of the phenyl rings. A bowl shape would also fit the observed STM contrast change, in which the brightness of the center at low biases is shifted to the phenyl rings with increasing bias. The adsorption heights derived from the relaxed DFT structure are in good agreement with the experimental ones and also show a slight bowl shape conformation (Table 3 and Figure 7b).

## Conclusion

We demonstrated the preparation of three wetting, regular self-assembled monolayers of Ru porphyrins on Ag(111) by using Ru-OEP and varying its packing density and substituents (on-surface conversion to Ru-TBP). In a comprehensive investigation of two distinct porphyrins, Ru-OEP and Ru-TBP on Ag(111), we examined the impact of coverage and substituents on adsorption geometry and self-assembly. Ru-OEP exhibits two different self-assemblies on Ag(111): a relaxed phase and a compressed phase. The relaxed phase unit cell consists of two molecules twisted by 31° in plane relative to each other, whereas no rotation between adjacent molecules is observed in the compressed phase. NIXSW measurements indicate that the metal center of Ru-OEP has a low adsorption height (2.45–2.50 Å), representative of a chemisorption of the metal center. Both NIXSW and NEXAFS data suggest a bowl-shaped conformation of Ru-OEP, corroborated by the relaxed structure observed in DFT calculations. The coverage-dependent phase change from relaxed to compressed phase induces a distortion in the conformation of the porphyrin, as evidenced by NIXSW measurements.

For Ru-TBP the porphyrin ring and the metal center exhibit a slightly higher adsorption height, as evidenced by NIXSW measurements and DFT calculations compared to Ru-OEP. However, the chemisorption character of Ru-TBP on Ag(111) is still obvious. A bowl-shaped conformation is indicated by NIXSW and DFT, with the phenyl rings pointing out of the surface plane. This is in accordance with the conformation of the macrocycle and the phenyl rings in the planarized Ru-TPP derivates, which was further confirmed with CO-modified tip nc-AFM measurements and simulations.^50^

Finally, notable disparities in the electronic structure of Ru-OEP and Ru-TBP were identified in the valence band and the unoccupied states, as well as in the work functions of the monolayer interfaces. Our thorough investigation thus provides a benchmark for utilizing monolayers of prototypical metalated octaethyl porphyrins and their high-temperature derivatives on planar, metal surfaces. The observed variations in the properties of the two porphyrins in this study are anticipated to have implications for various porphyrin functions, such as gas sensing or catalysis.

## Supporting Information Available

Additional STM, LEED, NEXAFS data (PDF) (PDF)
