# Supplementary material for: Octaethyl vs Tetrabenzo Functionalized Ru Porphyrins on Ag(111): Molecular Conformation, Self-Assembly and Electronic Structure
Source: J Phys Chem C Nanomater Interfaces. 2024 Dec 16;129(1):858–69. doi: 10.1021/acs.jpcc.4c06978 (PMC11726639; doi:10.1021/acs.jpcc.4c06978)
Supplement: Supplementary file 1 — jp4c06978_si_001.pdf [file jp4c06978_si_001.pdf]

Supporting Information

Octaethyl *vs.* Tetrabenzo Functionalized Ru Porphyrins on Ag(111): Molecular Conformation, Self-Assembly and Electronic Structure

Dennis Meier[a], Peter Knecht[a], Pablo Vezzoni Vicente[a], Fulden Eratam[b], Hongxiang Xu[a], Tien-Lin Lee[b], Alexander Generalov[c], Alexander Riss[a], Biao Yang[a], Francesco Allegretti[a], Peter Feulner[a], Joachim Reichert[a], Johannes V. Barth[a], Ari Paavo Seitsonen[d,e], David A. Duncan[b] and Anthoula C. Papageorgiou[a,f]**

[a] Technical University of Munich, TUM School of Natural Sciences, Physics Department E20, 85748 Garching, Germany

[b] Diamond Light Source, Didcot OX11 0QX, U.K.

[c] MAX IV Laboratory, Lund University, 22 484 Lund, Sweden

[d] Département de Chimie, École Normale Supérieure (ENS), 75005 Paris, France

[e] Université de recherche Paris-Sciences-et-Lettres, Sorbonne Université, Centre National de la Recherche Scientifique, 75005 Paris, France

[f] Laboratory of Physical Chemistry, Department of Chemistry, National and Kapodistrian University of Athens, Panepistimiopolis, 157 71 Athens, Greece

Table of Contents

Figure S1: Preparation of Ru-TBP 2
Figure S2: LEED of relaxed Ru-OEP phase 3
Figure S3: Bias dependency of Ru-OEP 3
Figure S4: LEED of compressed phase Ru-OEP on Ag(111) 4
Figure S5: Individual Ru-TBP molecules 4
Figure S6: Bias dependency of Ru-TBP 5
Figure S7: LEED of Ru-TBP on Ag(111) 5
Figure S8: Unit cell comparison of Ru-TBP on Ag(111) by LEED and STM 6
Figure S9: N K-edge NEXAFS of relaxed Ru-OEP 6

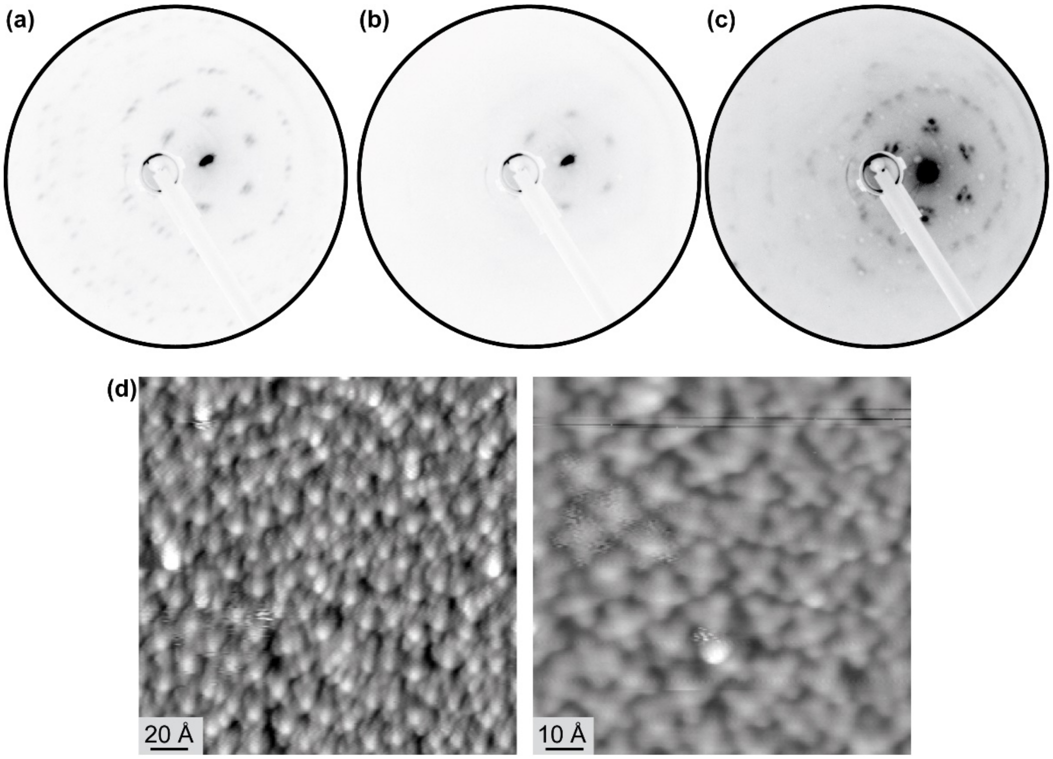

Figure S1. LEED of (a) compressed phase of Ru-OEP, (b) after annealing to 600 K, and (c) Ru-TBP prepared on a sample at 700 K. All LEED patterns were taken with an electron energy of 30 eV. (d) STM images of a monolayer of Ru-OEP annealed to approximately 600 K (left: -1768 mV, 80 pA, RT; right: 1768 mV, 110 pA, RT).

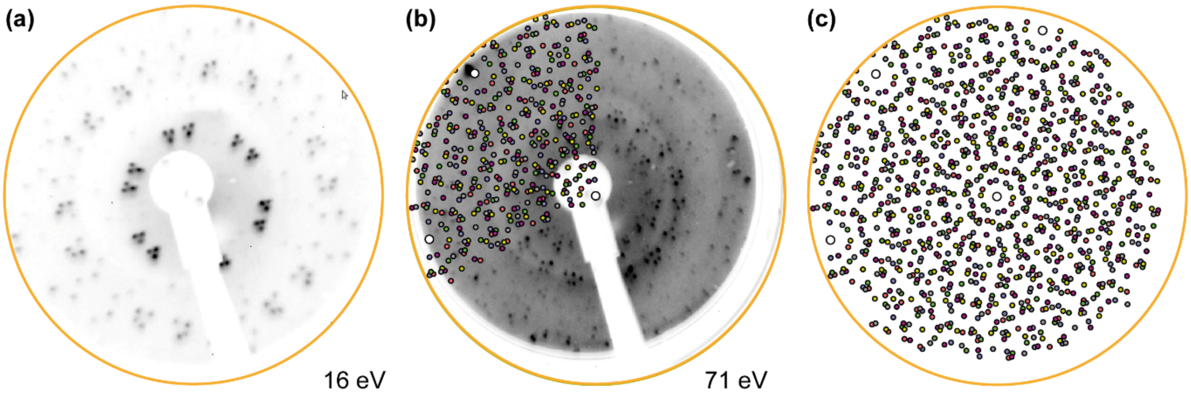

Figure S2. (a-b) LEED of relaxed phase Ru-OEP on Ag(111). On the top left of (b) the simulated pattern is overlayed for comparison. The respective electron energy is noted at the bottom right of the image. (c) Complete simulated pattern.

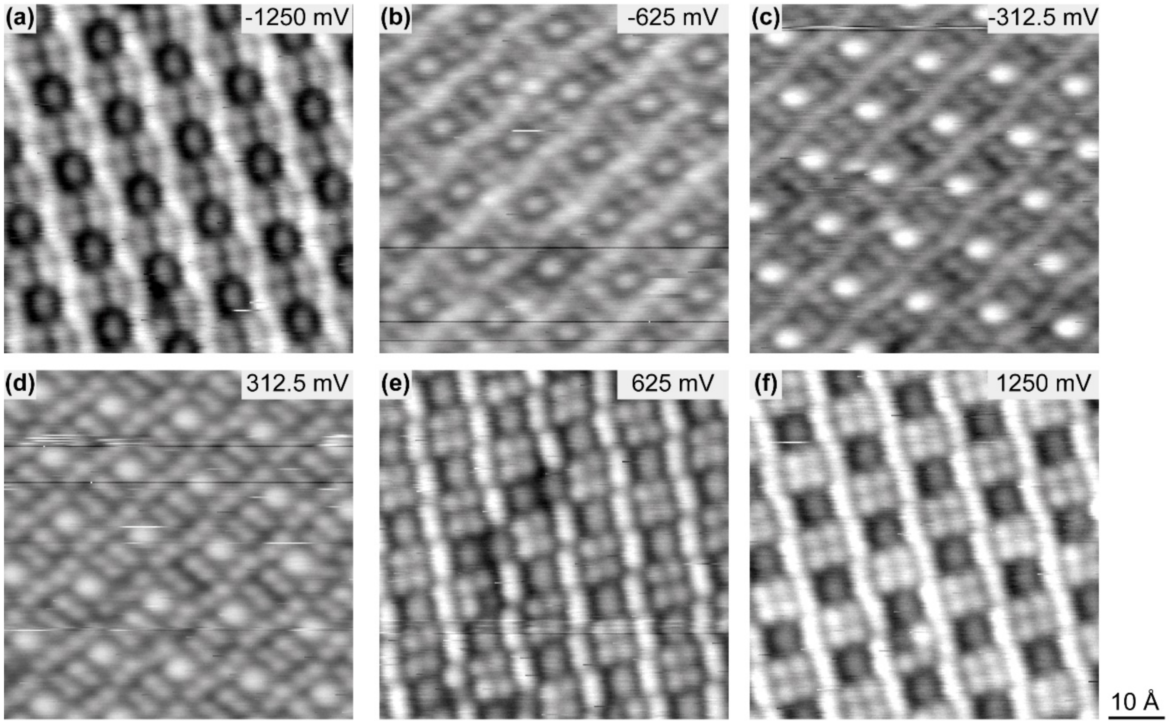

Figure S3. STM images of compressed phase of Ru-OEP on Ag(111) at different biases at rt (a: 30 pA; b: 20 pA; c: 20 pA; d: 50 pA; e: 60 pA; f: 40 pA). The positive and negative bias at 1250 mV shows a darker porphyrin center compared to the ethyl side chains. This is well matching with the valence band measurements, which show a state at 0.51 eV and no state at 1.25 eV beneath Fermi.

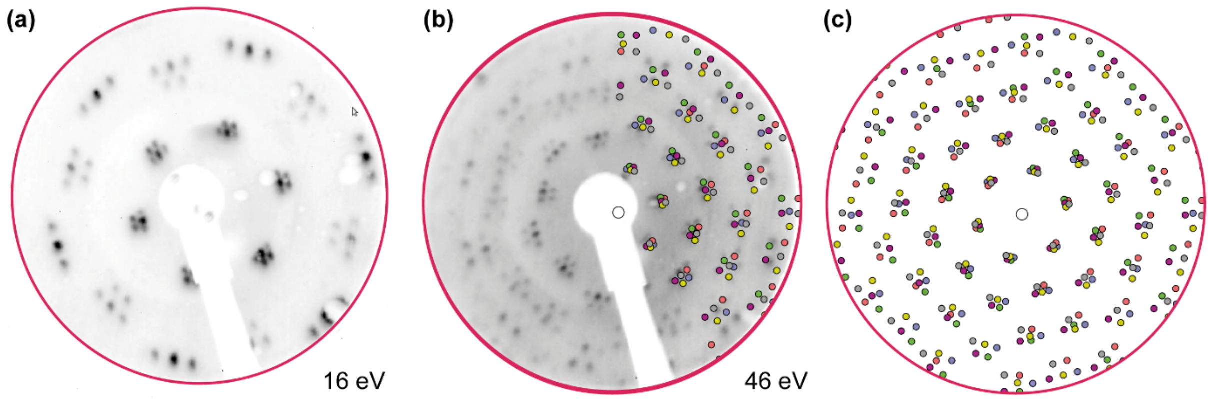

Figure S4. (a-b) LEED of the compressed phase Ru-OEP on Ag(111). On the top left of (b) the simulated pattern is overlayed for comparison. The respective electron energy is noted at the bottom right of the image. (c) Complete simulated pattern.

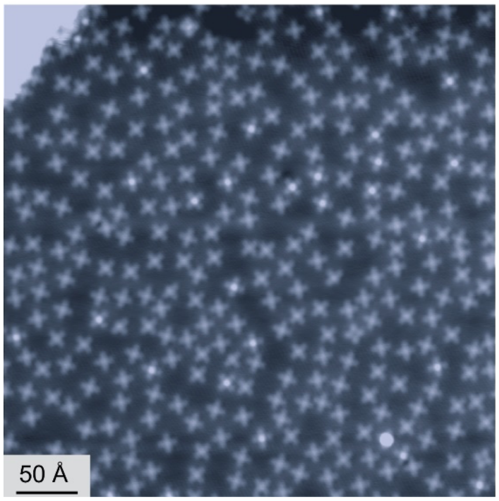

Figure S5. STM image of low coverage Ru-TBP on Ag(111) (50 pA, 200 mV, 6 K).

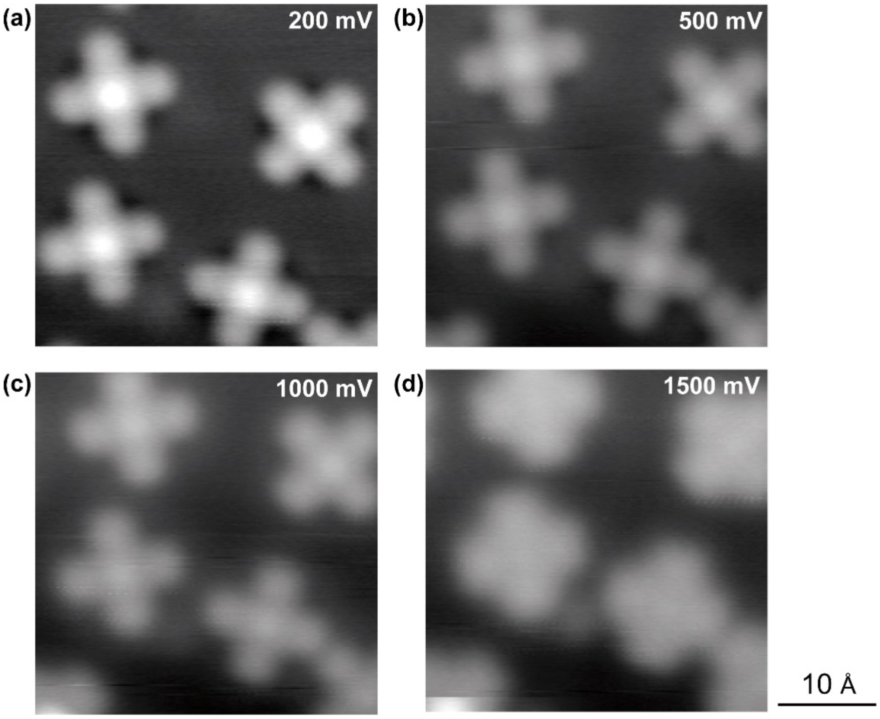

Figure S6. (a-d) STM images of Ru-TBP on Ag(111) at 5 K, captured at different biases as indicated in each image. All images were acquired at 20 pA.

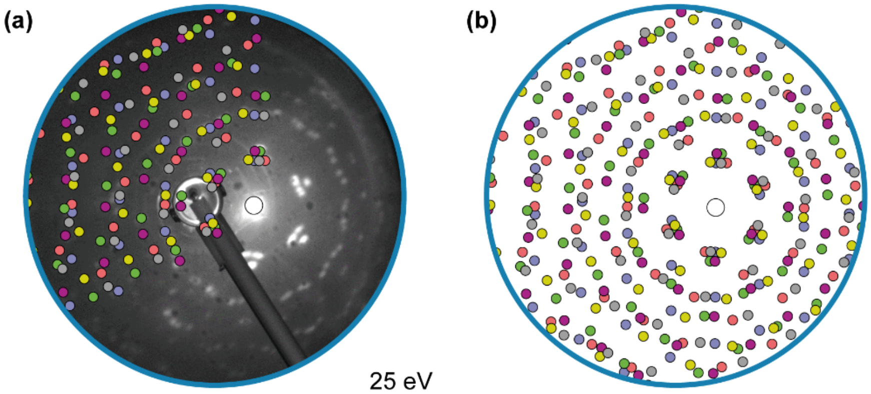

Figure S7. (a) LEED of Ru-TBP on Ag(111). On the top left the simulated pattern is overlayed for comparison. The respective electron energy is noted at the bottom right of the image. (c) Complete simulated pattern.

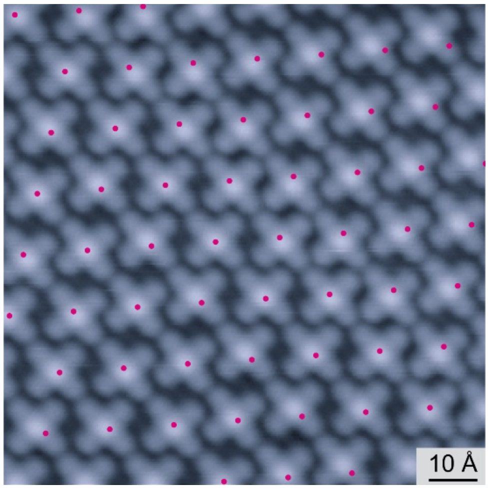

Figure S8: Position of Ru-TBP derived by LEED at 200 K (pink dots) overlaid over an STM image (50 pA, 200 mV, 5 K). Small deviations between the LEED positions and the STM image are observed. This is ascribed tentatively to the different acquisition temperature of the techniques.

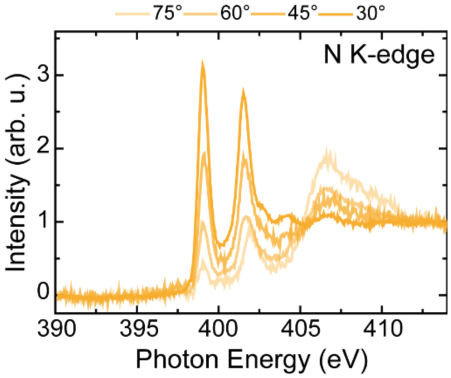

Figure S9. N K-edge NEXAFS measurements of the relaxed phase of Ru-OEP on Ag(111) acquired at four different incidence angles. The spectra exhibit a pronounced dichroism in the π* region comparable to the compressed phase of Ru-OEP on Ag(111).
